# New Genotypes and Phenotypes in Patients with 3 Subtypes of Waardenburg Syndrome Identified by Diagnostic Next-Generation Sequencing

**DOI:** 10.1155/2019/7143458

**Published:** 2019-02-27

**Authors:** Wu Li, Lingyun Mei, Hongsheng Chen, Xinzhang Cai, Yalan Liu, Meichao Men, Xue Zhong Liu, Denise Yan, Jie Ling, Yong Feng

**Affiliations:** ^1^Department of Otolaryngology, Xiangya Hospital, Central South University, 87 Xiangya Road, Changsha, Hunan, China; ^2^Province Key Laboratory of Otolaryngology Critical Diseases, Changsha, Hunan, China; ^3^Health Management Center, Xiangya Hospital, Central South University, 87 Xiangya Road, Changsha, Hunan, China; ^4^Department of Otolaryngology, University of Miami, Miller School of Medicine, Miami, USA; ^5^Dr. John T. Macdonald Foundation Department of Human Genetics, University of Miami, Miller School of Medicine, Miami, FL 33136, USA; ^6^Institute of Molecular Precision Medicine, Xiangya Hospital, Central South University, Changsha, Hunan, China

## Abstract

**Background:**

Waardenburg syndrome (WS) is one of the most common forms of syndromic deafness with heterogeneity of loci and alleles and variable expressivity of clinical features.

**Methods:**

The technology of single-nucleotide variants (SNV) and copy number variation (CNV) detection was developed to investigate the genotype spectrum of WS in a Chinese population.

**Results:**

Ninety WS patients and 24 additional family members were recruited for the study. Fourteen mutations had not been previously reported, including c.808C>G, c.117C>A, c.152T>G, c.803G>T, c.793-3T >G, and c.801delT on *PAX3*; c.642_650delAAG on *MITF*; c.122G>T and c.127C>T on *SOX10*; c.230C>G and c.365C>T on *SNAI2*; and c.481A>G, c.1018C>G, and c.1015C>T on *EDNRB*. Three CNVs were de novo and first reported in our study. Five *EDNRB* variants were associated with WS type 1 in the heterozygous state for the first time, with a detection rate of 22.2%. Freckles occur only in WS type 2. Yellow hair, amblyopia, congenital ptosis, narrow palpebral fissures, and pigmentation spots are rare and unique symptoms in WS patients from China.

**Conclusions:**

*EDNRB* should be considered as another prevalent pathogenic gene in WS type 1. Our study expanded the genotype and phenotype spectrum of WS, and diagnostic next-generation sequencing is promising for WS.

## 1. Introduction

Waardenburg syndrome (WS) is a rare genetic disorder with a reported frequency estimated to be approximately 1 : 40000 in the general population but is present in approximately 3% of all patients with congenital deafness [1, 2]. The disorder is characterized by the presence of pigmentation abnormalities, including depigmented patches of the skin and hair, heterochromia iridis, and sensorineural hearing impairment. Dystopia canthorum, musculoskeletal abnormalities of the limbs, or Hirschsprung disease, are used for the clinical classification. Four subtypes of WS have been described based on the clinical manifestations. Six genes are involved in this inherited disorder, including *PAX3* (encoding the paired box 3 transcription factor), *MITF* (microphthalmia-associated transcription factor), *SOX10* (encoding the Sry box 10 transcription factor), *EDNRB* (endothelin receptor type B), *EDN3* (endothelin 3), and *SNAI2* (SNAIL homolog 2). WS type 1 (OMIM # 193500) was first described by Waardenburg [3]. Dystopia canthorum, an outward displacement of the inner canthus of the eyes, is the most penetrant feature of WS type 1. *PAX3* mutations account for the majority of WS type 1 cases. Features of WS type 2 (OMIM #) show marked interfamilial and intrafamilial variability. There are 3 genes linked to WS type 2, namely, *MITF*, *SOX10*, and *EDNRB*. However, pathogenic genes cannot be detected in 70% of WS type 2 cases [4]. WS type 3 is associated with limb deformities together with the symptoms observed in type 1. *PAX3* mutations have also been found in the heterozygous or homozygous state in WS type 3 (OMIM # 148820) [5]. WS type 4 (OMIM # 277580), also called the Shah-Waardenburg syndrome, is characterized by the association of deafness, depigmentation, and intestinal aganglionosis (called Hirschsprung disease (HD)). The endothelin pathway (endothelin 3 (*EDN3*), endothelin receptor type B (*EDNRB*), and Sry box 10 (*SOX10*)) was found to be involved in WS type 4 [6, 7].

It has been determined that pathogenic mutations were not and still cannot be detected in a considerable number of WS cases. Other types of variations may exist, given the limitations of conventional detection technology. Copy number variation (CNV) is a new topic of increasing interest in genetic research. In addition, CNV has been reported to be associated with WS [8–10]. Recently, one study addressed the molecular etiology investigation of WS in individuals mostly from southeastern Brazil by sequential Sanger sequencing of all coding exons of the 6 WS-associated genes, followed by CNV detection by multiplex ligation-dependent probe amplification (MLPA) of the *PAX3*, *MITF*, and *SOX10* genes, and revealed novel pathogenic mutations [11]. Traditional sequencing methods are designed for point mutation detection without considering the possibility of CNV. Exon capture sequencing for simultaneous SNV and CNV detection in WS has been developed in our study.

To date, there is no large cohort on the genotype and phenotype spectrum of WS patients from China. In this study, we aimed to investigate the genetic etiology and phenotype differences using target exon capture of 6 known causative genes in WS.

## 2. Materials and Methods

### 2.1. Patients and Family Members

**Figure 1:**
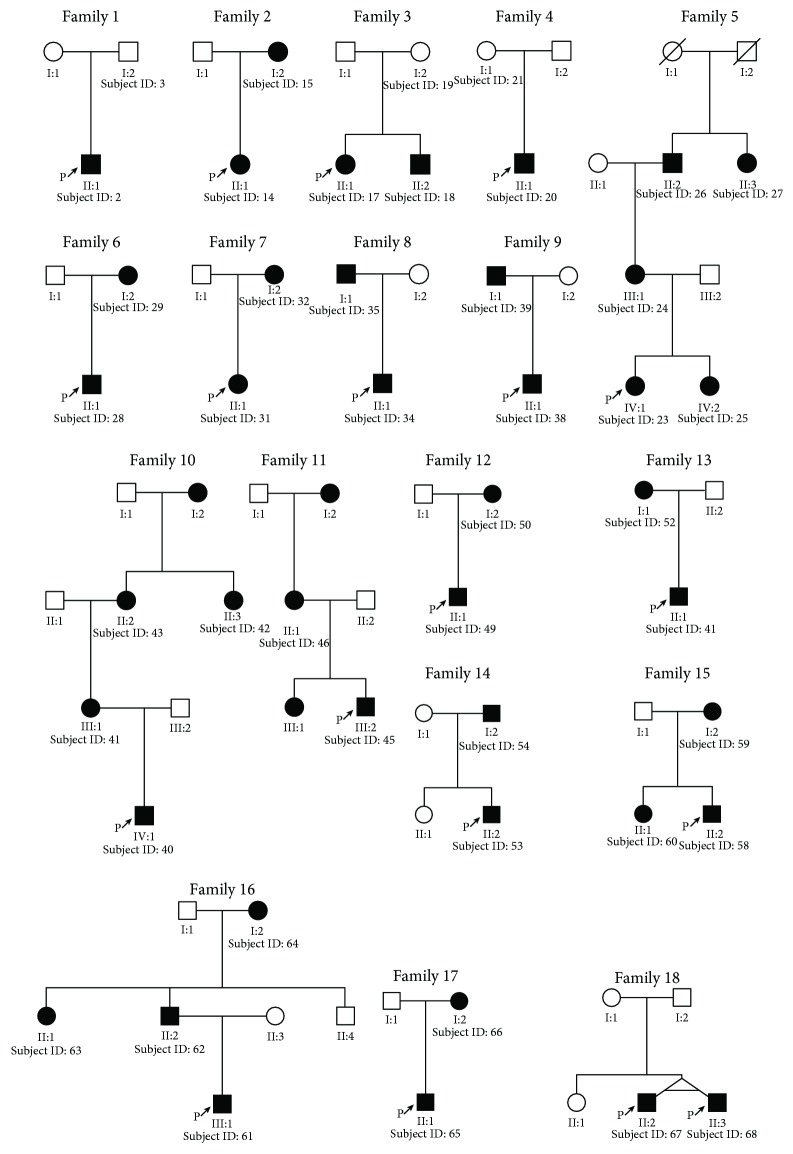
Eighteen WS families with at least 2 DNA samples and clinical information collected in the study. Family ID and Subject ID were added to the individuals with DNA samples. A table with all data for family cases was shown in Table 1.

The patients diagnosed with WS were examined occasionally at the Otology Clinic and training schools for deaf and mute individuals in China from September 2006 to February 2018. There were 114 participants, including sporadic WS cases and 18 families (Figure 1, Table 1). All the WS patients were clinically evaluated by at least one otologist. The clinical signs and symptoms of the 90 patients diagnosed with WS are presented in Table S1. Two hundred randomly selected normal hearing individuals were included in this study. Blood samples (4-6 ml) were extracted from the peripheral veins of all the participants for DNA extraction. The ethics committee of Xiangya Hospital, Central South University, had approved this study, and signed informed consents were obtained from each of the subjects or their guardians.

### 2.2. Clinical Evaluation

A comprehensive clinical history was collected by questionnaire and telephone inquiry. The audiological examinations consisted of otoscopy, pure-tone audiometry (PTA) or auditory brainstem response (ABR), immittance, and distortion product otoacoustic emission (DPOAE). Special attention was given to the color of the skin, hair, and irises and other developmental defects. Assessment of dystopia canthorum on the basis of ocular measurements was described by Farrer et al. [12].

### 2.3. Exon Capture Sequencing for Simultaneous SNV and CNV Detection in WS

**Figure 2:**
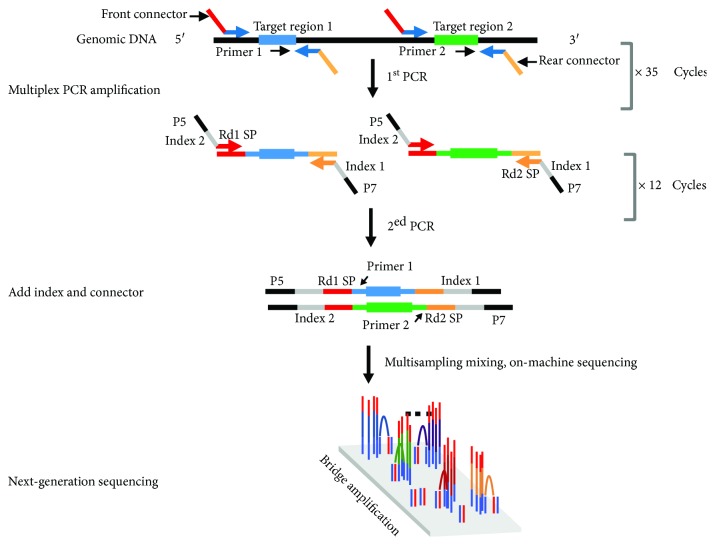
Multiple PCR target enrichment and next-generation Sequencing of WS-related genes.

The primer sequences for 6 WS-related genes were designed for the target regions (Table S2), and the regions of interest were captured and enriched. PCR amplification was divided into two rounds. The first round of PCR amplification was to amplify the 6 relevant WS genes. The amplification region includes the promoter regions (~500 bp), 5′untranslated region (5′UTR), coding regions, splice sites (~8 bp), and 3′untranslated region (3′UTR) (Table 2). The multiplex PCR amplification system can amplify 20-30 gene fragments at the same time. Each sample requires approximately 4 multiplex PCRs to complete the first round of enrichment. The second round of PCR added the 4 multiplex PCR products above into a mixture by amplifying the universal sequence. Indexes were added to distinguish between different samples. After these two rounds of amplification were finished, the WS sequencing library was constructed. Bidirectional sequencing validation of the target segments was performed by 2x 250 bp sequencing with an Illumina MiSeq Sequencer. The average effective sequencing depth for each sample was 300x, with all bases having greater than 20x sequencing depth (Figure 2)

The CNV detection technique utilizes ligase to hybridize and ligate the region of interest. Then, different lengths of the ligated products corresponding to the loci were obtained by introducing nonspecific sequences of different lengths to the ends of the ligation probes and performing a ligation reaction. The PCR product was amplified by fluorescently labeled universal primers. The amplified products were separated by fluorescence capillary electrophoresis and analyzed by electrophoresis. The peak height at each site was analyzed

### 2.4. Bioinformatics Analysis

After mapping WS-related gene sequences to the DNA samples, the results were aligned with the reference genome. Sequencing data quality was assessed through the sequencing depth of each fragment of each sample. Inconsistent sites were detected by comparison with the reference genome, which is called SNV calling. Then, SNVs were functionally annotated and functional candidate sites were determined.

### 2.5. Sanger Sequencing for the Segregation of Candidate Variants

DNA samples of affected siblings and their available first-degree relatives were collected for segregation analysis of candidate variants. For validation and segregation analyses, the primers were designed to amplify the regions flanking the variant as previously described [4, 13]. Segregation analysis by Sanger sequencing was also performed in 200 ethnically matched individuals with normal hearing. These included variants annotated as nonsense mutations, splicing mutations disrupting either a splice donor or acceptor site, frameshift or non-frameshift-causing InDels, and missense mutations predicted as damaging by at least one of the following methods: SIFT, Polyphen2_HVAR, Polyphen2_HDIV, MutationTaster, and CADD [14–16]. Nonsynonymous SNVs with a SIFT score < 0.05, Polyphen2_HVAR score ≥ 0.047, Polyphen2_HDIV ≥ 0.0453, MutationTaster score > 0.85, or CADD score > 15 were considered significantly deleterious. To sort potentially deleterious variants from benign polymorphisms, Perl scripts were used to filter the SNVs against those in the 1000 Genomes and esp6500si_all databases. We also tested all the variants for their allele frequencies in the Exome Aggregation Consortium (ExAC) (http://exac.broadinstitute.org/) to further support the pathogenicity of the new variants detected. The SNV recorded in the population database with a minor allele frequency of <1/100000 in the population from the database was considered disease causing and therefore remained.

## 3. Results

**Figure 3:**
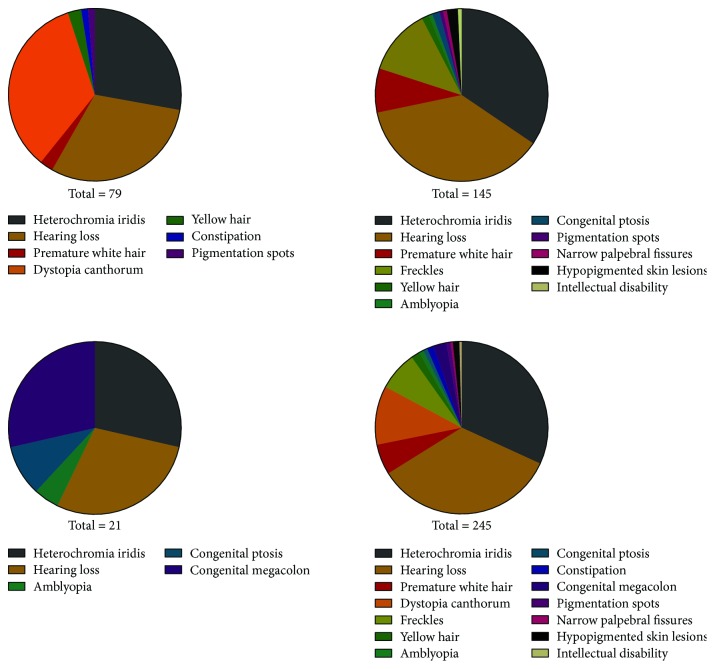
The proportion of phenotypes detected in WS types 1, 2, and 4. Dystopia canthorum was the most frequent sign in WS type 1 (100%, 27/27), followed by sensorineural hearing loss (88.9%, 24/27), heterochromia iridis (81.5%, 22/27), hair hypopigmentation (14.8%, 4/27), constipation (1/27, 3.7%), and pigmentation spots (1/27, 3.7%). In WS type 2, sensorineural deafness (94.7%, 54/57) and heterochromia iridis (87.7%, 50/57) were still the most common clinical signs and symptoms, followed by freckles (31.6%, 18/57), hair hypopigmentation (24.6%, 14/57), hypopigmented skin lesions (5.3%, 3/57), and congenital ptosis (3.5%, 2/57). Amblyopia (1.8%, 1/57), congenital ptosis (1.8%, 1/57), and narrow palpebral fissures (1.8%, 1/57) are rare and unique symptoms in WS type 2 in the Chinese population.

Most proteins associated with the known WS genes are involved in melanocyte migration and neural crest and inner ear cell development. The clinical symptoms of WS result from neural crest embryonic cell defects. Sensorineural hearing loss is one of the most common signs and symptoms (93.3%, 84/90) in the 90 cases meeting the diagnostic criteria for WS. Auditory function is variable within and between families, ranging from normal to profound deafness. Bilateral deafness is much more common than unilateral, as only one case in our study was found to have unilateral hearing loss. The reported prevalence of temporal bone abnormalities varies from 0 to 50% [17]. Vestibular aqueduct dilatation, together with Waardenburg syndrome, was discovered in 2 cases (Subjects 113 and 114) in our study. No vestibular dysfunctions were found in any of the participants. A white forelock was present in 15.6% (14/90) of all 90 cases. Interestingly, 4 patients of Chinese Han race WS patients presented with yellow hair before reaching 3 years old. All reported hair color in WS patients was prematurely graying and turning white, but the normal hair color in the Chinese Han race is black. Hypoplastic iridis, particularly brilliant blue eyes, was present in 86.7% (78/90) of WS patients. The heterochromia was observed to be complete or segmental. The following prevalent phenotype included freckles on the face (20.0%, 18/90), which seems to be a special clinical sign in the Chinese population, given its high occurrence rate. The rate of hypopigmented skin lesions was more rare than that in other populations [18], with only 3 in 90 cases (3.3%) being found. Pigmentation spots (2.2%, 2/90) on the skin might be a special subtype of skin pigmentation disturbances. Other rare and unreported phenotypes, including amblyopia, congenital ptosis, and narrow palpebral fissures, were recorded to accompany WS, but no clear evidence has been found showing that these phenotypes relate to neural crest embryonic cell defects at present (Figure 3).

**Figure 4:**
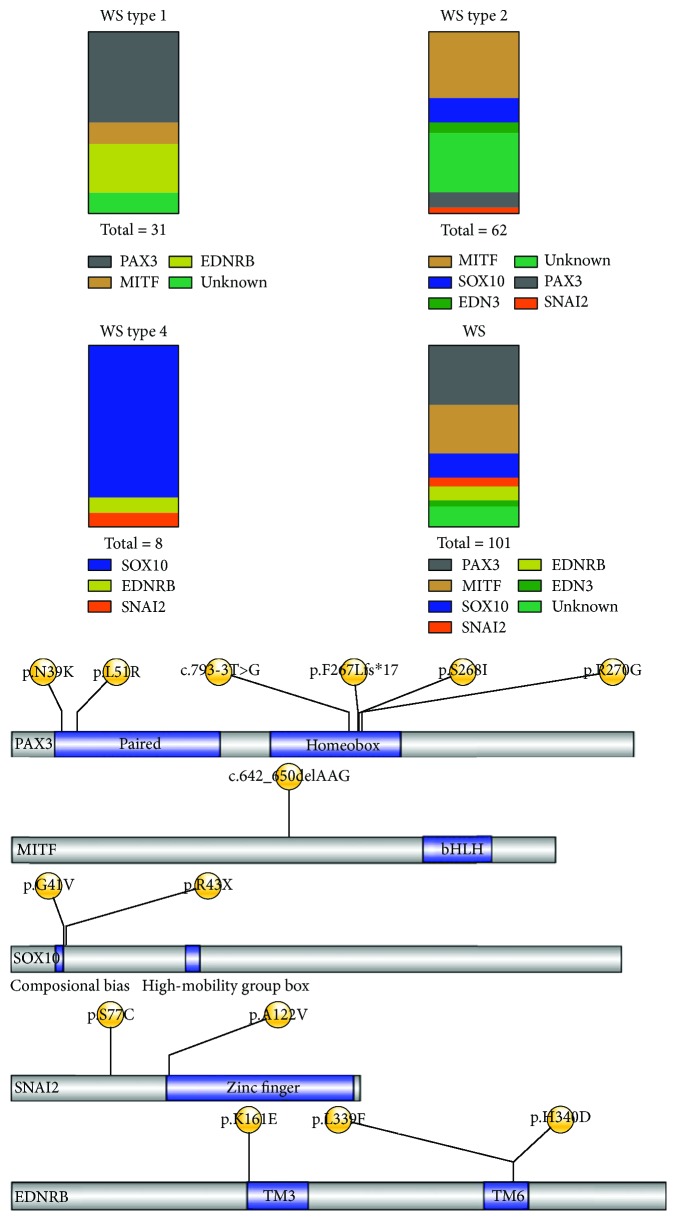
The proportion of genotypes detected in WS types 1, 2, and 4 and the position in the protein domains of the new SNVs detected in our study.

The subtypes of WS were defined on the basis of the presence or absence of additional symptoms. WS type 1 was characterized by dystopia canthorum and WS type 2 with no additional features. Type 4 was also called Shah-Waardenburg syndrome, Hirschsprung disease included. Twenty-seven cases were diagnosed as WS type 1, 57 as WS type 2, and 6 as WS type 4. There were no WS type 3 cases recruited. WS types 1 and 2 were more frequent than type 4. The diagnostic next-generation sequencing in all 114 participants revealed 119 variants (Table S3); however, only 90 cases were diagnosed as WS according to the widely accepted diagnostic criteria [19]. Of the 90 WS patients, 49 were considered causative in WS and recorded in the Human Gene Mutation Database (HGMD) with a known disease mutation detection rate of 53.3% (49/90). Fourteen unreported mutations were detected and selected for further bioinformatics pathogenicity analysis (Table 3). Three CNVs discovered are listed separately in Table 4.  Figure 4 shows the position in the protein domains of the new SNVs detected in our study. WS type 2 is mainly caused by heterozygous pathogenic variants in genes *MITF* (24/57, 42.1%), *PAX3* (10/57, 17.5%), and *SOX10* (9/57, 15.8%). Homozygous deletions in *SNAI2* were also reported to associate with WS type 2 cases [20]. One variant was detected in our research, which indicated that *SNAI2* mutations seem to be rare in WS type 2 among Eastern and Western populations. In contrast with the reported findings, *EDN3* mutations were also detected in the WS type 2 cases (Subjects 72, 76, and 104) in our study. Even after extensive analysis of the 6 known genes, a percentage of the WS type 1 and type 2 cases, 14.8% (4/27) and 26.3% (15/57), respectively, remained molecularly unexplained (Figure 4). No WS type 3 cases were recruited in our study. The *EDNRB*, *EDN3*, and *SOX10* mutations [21, 22] are related to WS type 4, and close to 80% of the WS type 4 cases were found to be caused by mutations in *SOX10* in our study.

Two CNVs and 3 SNVs, including c.110_219del110bp in *MITF*, the whole-gene deletion in *SOX10*, c.801delT on *PAX3*, c.642_650delAAG on *MITF*, and c.127C>T on *SOX10*, resulted in a truncated protein with a premature termination, which were loss-of-function (LoF) mutations and therefore considered to be cause of disease. The last CNV meant a duplicate sequence from promoter 2 to exon 1 on *MITF*, which might be one of the reasons for a gain-of-function (GoF) mechanism. Therefore, protein function is activated. The expression or degradation of proteins resulted in increased protein dosage. All the remaining missense substitutions were selected for pathogenicity prediction and population frequency, and the forecast results and data are shown in Table 3.

## 4. Discussion

WS is a genetic disorder with locus heterogeneity and variable expressivity of clinical features [23]. WS type 1 and type 2 are conspicuously differentiated by the presence or absence of dystopia canthorum among populations [18]. In this present study, we have identified mutations in *PAX3* in cases (Subjects 2, 4, 6, 49, 51, 54, 90, 91, 98, and 104) without dystopia canthorum, typical WS type 2 characteristic; conversely, Subjects 67, 68, and 110 with the clinical feature of WS type 1 (with the presence of dystopia canthorum) were found to carry *MITF* variants. Thus, it is difficult to establish a link between a genotype and a classical WS phenotype in our cohort. This implies that other factors including interactions between genes, gene-environment interactions, and ethnic background may modulate WS phenotypes.

Overall, 55.5% (15/27) of the cases of WS type 1 are caused by pathogenic variants in *PAX3*. A recent study suggests that homozygous mutation in *EDNRB* can cause the WS type 1 phenotype [24], whereas in our study, heterozygous mutations in *EDNRB* (Subjects 48, 65, 66, 85, 108, and 111) were related to WS type 1 for the first time, with a detection rate of 22.2% (6/27).

Six pathogenic genes have been associated with the clinical manifestations so far. Overall, 81 pathogenic or likely causative variants were associated with WS in our sample of 90 WS patients, including the probands and their available family members, and consisting of 27 variants located in *MITF* (33.3%, 27/81), 25 in *PAX3* (30.9%, 25/81), 15 in *SOX10* (18.5%, 15/81), 9 in *EDNRB* (11.1%, 9/81), 3 in *EDN3* (3.7%, 3/81), and 2 in *SNAI2* (2.5%, 2/81).

In the group of 27 participants with WS type 1, 15 were found to carry mutations in *PAX3*, which corresponds to a detection rate of 55.5% (15/27). In the review by Pingault et al. [18], it is stated that 90% of WS type 1 patients have mutations in the *PAX3* gene. On the other hand, in the sample from Caucasian [25], mutations in *PAX3* were detected in 33.6% of 119 patients with clinical suspicion of WS. In the latter study by Bocángel et al. [11], 11 variants were located in *PAX3* (57.9%). Our data were very similar to the results of Bocángel et al. [11]. Genetic analyses in a study by Morimoto et al. [24] revealed that the proband had a missense mutation (p.R319W) in the *EDNRB* gene. In our study, 4 *PAX3*-negative WS type 1 patients (Subjects 48, 85, 108, and 111) were found to carry at least one novel *EDNRB* heterozygous mutation, suggesting that *EDNRB* is the second most prevalent pathogenic gene and should be considered for screening analysis in WS type 1 patients.

Three mutations in *MITF* were linked to WS type 1, and 10 variants in *PAX3* were related to WS type 2. Dystopia canthorum is considered as the most reliable feature for WS type 1 classification due to its very high penetrance [19]. In fact, Asian individuals generally have a wider and lower nasal root than European and American individuals, suggesting that the classification criteria cannot be applied to all populations and molecular genetic testing may be a complementary tool for establishing the diagnosis. In the group of 57 individuals with WS type 2, mutations in 4 other causative subtype genes are 51.1% (24/47) in *MITF*, 19.1% (9/47) in *SOX10*, 6.4% (3/47) in *EDN3*, and 2.0% (1/47) in *SNAI2*. Compared to Pingault et al.' s review [18], in which the overall detection rate in WS type 2 is approximately 50%, our detection technology (73.7%, 42/57) has absolute advantages. *SNAI2* and *EDN3* mutations in WS type 2 are not observed frequently, which is similar to our data. Three CNVs overlapping the *MITF* and *SOX10* genes were found in 3 patients (Subjects 9, 22, and 33).

It is also remarkable that known disease mutations were inherited from unaffected fathers in Families 1 (Subjects 2 and 3), 3 (Subjects 17, 18, and 19), and 4 (Subjects 20 and 21). The c.238C>G in *PAX3* is a known disease-causing mutation. It is puzzling is that there are no WS-related clinical manifestations appearing in Subject 3 who harbors the same mutation. The results found in our study greatly expanded the database of hotspot and novel mutations in WS types 1, 2, and 4. PAX3 is a transcription factor expressed during embryonic development [26] and four structural motifs, paired domain, octapeptide sequence, homeodomain, and a Pro-Ser-Thr-rich COOH terminus, were included in PAX3. The 6 mutations in *PAX3*, c.808C>G, c.117C>A, c.152T>G, c.793-3T>G, c.803G>T, and c.801delT, are located in the highly conserved domain of PAX3. Alterations in this domain may lead to a decrease in DNA binding affinity or a change in DNA binding specificity. MITF is also a transcription factor. A basic helix-loop-helix zipper motif is vital for the survival and development of melanocytes. The *MITF* mutation, c.642_650 delAAG, results in a premature termination codon, and the mutant protein is void of functional domains. The variant likely results in disease through the mechanism of haploinsufficiency. SOX10 is a member of the group E SOX genes. A central high-mobility group (HMG) domain and a C-terminal transactivation domain were included in the protein [27]. In this study, we identified two novel *SOX10* mutations, c.122G>T and c.127C>T, as associated with WS type 2 in the Chinese population. As described in Table 3, c.122G>T in *SOX10* was not considered causative due its frequency, which was >1/10000 in the population database of ExAC_EAS. In addition, c.122G>T in *SOX10* can be observed in Figure 4 that this mutation affects an amino acid residue located outside the high-mobility group box domain of SOX10. More specifically, the c.122G>T variant in *SOX10* (Subjects 20 and 21, Figure 1) was inherited from the unaffected mother. The c.127C>T variant on *SOX10* was regarded as likely causative, because of its in silico predictions of pathogenicity and it is absent in 200 normal controls and with no frequency in population databases.

The SNAIL-related zinc-finger transcription factor SNAI2 (Slug) is a member of the SNAIL family of zinc-finger TFs that share an evolutionarily conserved role in mesoderm formation [28]. SNAI2 is expressed in migratory neural crest cells (NCCs) and is indispensable for melanoblast survival or migration. We had detected 2 variants (c.230C>G and c.365C>T) in *SNAI2* in the cohort of WS participants that were not considered causative due to their frequency (>1/10000) in the population database. Therefore, *SNAI2* has a minor involvement in WS in the Chinese population. The endothelins are a group of three peptides (ET1, ET2, and ET3) [29]. In vertebrates, Ednrb (encoding the endothelin receptor type B (ETB)) is first expressed at the dorsal tip of the neural tube, then in NCCs in both dorsoventral and dorsolateral pathways [30]. The c.469A>G, c.553G>A, c.481A>G, c.1015C>T, and c.1018C>G mutations in *EDNRB* were detected in patients with WS type 1 in the present study in the Chinese population for the first time. Six cases were found to carry c.49G>A in *EDN3* that has been linked to WS type 4 in the HGMD database.

The clinical manifestations of WS vary widely between different populations. In the present study, heterochromia iridis and sensorineural deafness were the most frequent features of both WS type 1 and WS type 2. Freckles were not observed in WS type 1 patients but were present in 31.6% (18/57) of type 2 patients, which might be a preidentified indicator between types 1 and 2 in Chinese populations. The present study and those of Silan et al. [31] and Tamayo et al. [32] were the primary screening programs in the institutionalized deaf populations in China, Turkey, and Colombia. In general, the distribution of WS type 2 is more common than that of type 1, which was in agreement with Silan et al. [31] and Tamayo et al. [32]. However, the majority of reports [33] presented more cases of WS type 1. We were looking for WS cases in hospitals or schools for deaf and mute individuals. Hearing loss is more common in WS type 2, which might be the reason resulting in the consequence. A white forelock was reported and estimated to be present in at least one-third of both WS type 1 and 2 cases [18]. However, the proportion of this phenotype in our population was much lower, which is different from the deaf populations in Turkey [31] and Colombia [32] and could be considered a difference among different populations. Ethnic migration may be the reason underlining this difference. Hypopigmented and depigmented patches on the skin can also be seen in previous reports from other populations [31, 32, 34]. Other associated symptoms described, such as cleft/lip palate, spina bifida, and musculoskeletal anomalies, were not found in the present study. In contrast, some specific signs recorded in our study are rare and were not reported in other populations, such as yellow hair, amblyopia, congenital ptosis, and narrow palpebral fissures.

Three cases (Subjects 70, 72, and 100) with genotypes linked to WS type 4 have type 2 phenotype characteristics in the present study. The observation of different phenotypes in Family 2 with the same mutations may be due to heterochromia penetrance associated with a *SOX10* mutation (Subjects 14 and 15). The presence versus absence of WS features in *MITF* (Subjects 28 and 29, Family 6) also argues for the influence of the genetic background, which supports the hypothesis that there is an interplay between genetic and environmental factors.

Wildhardt et al. [25] had screened *PAX3*, *MITF,* and *SOX10* CNVs by MLPA and detected *PAX3* and *MITF* in the search for pathogenic variants. However, other associated genes were not included in their cohort. Bocángel et al. carried out the molecular investigation of WS by sequential Sanger sequencing of all 6 coding exons; then, CNV detection by MLPA of *PAX3*, *MITF*, and *SOX10* genes in selected cases followed [11]. Detection by repeated Sanger sequencing would be time-consuming and expensive. Captured sequencing, also called diagnostic next-generation sequencing, has specifically targeted regions. Compared with whole-exome sequencing, the cost is greatly reduced and the depth of coverage can reach to 300x. The target region capture system in our study was designed to amplify and detect 6 WS pathogenic genes in one reaction system. The observations in our study have also proved that this technology would be a promising tool to identify the molecular etiology in WS. However, the molecular etiology of a variable fraction of cases remained unexplained [18, 35], indicating that noncoding regions should also be included for molecular analysis. Moreover, it is possible that novel disease-causing genes are implicated in WS. Zazo Seco et al. [36] identified mutations in *KITLG* in WS families. Whole-exome sequencing and even whole-genome sequencing will fully reveal candidate and novel genes in molecularly unexplained cases of WS.

## Figures and Tables

**Table 1:** All data for family cases.

Family ID	1	2	3	4	5	6	7	8	9	10	11	12	13	14	15	16	17		18
Subject ID	2.3.	14.15.	17.18.19.	20.21.	23.24.25.26.27.	28.29.	31.32.	34.35.	38.39.	40.41.42.43.	45.46.	49.50.	51.52.	53.54.	58.59.60.	61.62.63.64.	65.66.		67.68.
Gene	*PAX3*	*SOX10*	*SOX10*	*SOX10*	*MITF*	*MITF*	NA	*PAX3*	*PAX3*	*MITF*	NA	*PAX3*	*PAX3*	*PAX3*	NA	*MITF*	*PAX3*	*EDNRB*	*MITF*
Mutation	c.238C>G	c.743_744delAG	c.254G>A	c.122G>T	c.763C>T	c.328 C>T	—	c.784C>T	c.232G>A	c.944_946del	—	c.808C>G	c.117C>A	c.452-2A>G	—	c.1013+1G>A	c.793-3T>G	c.469A>G	c.1022_1023delTC
Protein mutation	p.H80D	p. E248Afs^∗^32	p.W85^∗^	p.G41V	p. R255^∗^	p.R110^∗^	—	p.R262^∗^	p.V78M	NA	—	,p.R270G	p.N39K	—	—	NA	NA	p.I157V	p.L341Rfs^∗^18

NA in gene: the molecular etiology of cases that remained unexplained. NA in protein mutation: protein changes that cannot be described.

**Table 2:** WS-associated genes and their PCR target regions.

Gene name	NM_accession	Exon number	Gene length	mRNA length	Exon length
*PAX3*	NM_181457.3	8	97284	2032	478 (268)|coding+3′UTR,215,166,206,135,130,236,466|coding+5′UTR
*SNAI2*	NM_003068.4	3	3764	2112	1312 (183)|coding+3 ′UTR,546,254|coding+5′UTR
*MITF*	NM_000248.3	9	31738	4472	156|coding+5′UTR,228,84,96,118,75,76,148,3491 (402)|coding+3′UTR
*EDNRB*	NM_000115.3	8	80049	4282	2854 (136)|coding+3′UTR,109,134,150,205,113,534|coding+5′UTR,183|5′UTR
*SOX10*	NM_006941.3	4	12221	2862	1887 (705)|coding+3′UTR,269,512|coding+5′UTR,194|5′UTR
*EDN3*	NM_207034.1	5	25549	2619	421|coding+5′UTR,313,177,46,1662 (129)|coding+3′UTR

**Table 3:** Summary of the new SNV results of the molecular screening of WS-related genes, including location of mutations, pathogenicity predictions, and population data.

Subject ID	WS type	Chromosome location	Gene	Mutation	Protein mutation	Pathogenicity prediction					Population frequency			Inheritance status
						SIFT_pred	Polyphen2_HVAR_pred	Polyphen2_HDIV_pred	MutationTaster_pred	CADD13_PHRED	1000g_EAS	ExAC_EAS	esp6500si_all	
49	2	Chr2(q36.1)	*PAX3*	c.808C>G	p.R270G	NA	D	NA.	D	32	—	—	—	Novel
51	2	Chr2(q36.1)	*PAX3*	c.117C>A	p.N39K	T	D	D	D	25.9	—	—	—	Novel
109	1	Chr2(q36.1)	*PAX3*	c.808C>G	p.R270G	D	D	B	D	32	—	—	—	Novel
112	1	Chr2(q36.1)	*PAX3*	c.152T>G	p.L51R	D	D	D	D	29.5	—	—	—	*De novo*
65,66	1	Chr2(q36.1)	*PAX3*	c.793-3T>G	NA	NA	NA	NA	D	12.22	—	—	—	Novel
113,114	1	Chr2(q36.1)	*PAX3*	c.803G>T	p.S268I	D	D	D	D	33	—	—	—	*De novo*
113,114	1	Chr2(q36.1)	*PAX3*	c.801delT	p. F267Lfs^∗^17	T	NA	P	NA	NA	—	—	—	*De novo*
7,8	2	Chr3(P13)	*MITF*	c.642_650delAAG	NA	NA	NA	NA	NA	NA	—	—	—	*De novo*
20	4	Chr22(q13.1)	*SOX10*	c.122G>T	p.G41V	T	B	B	D	15.9	0.0109	0.0082	0.000077	*De novo*
102	4	Chr22(q13.1)	*SOX10*	c.127C>T	p.R43X	D	NA	B	A	35	—	—	—	*De novo*
76	2	Chr22(q13.1)	*SOX10*	c.122G>T	p.G41V	D	B	D	D	15.9	0.0109	0.0082	0.000077	*De novo*
76	2	Chr8(q11..21)	*SNAI2*	c.230C>G	p. S77C	D	B	P	D	21.3	0.004	0.0045	—	*De novo*
17	2	Chr8(q11..21)	*SNAI2*	c.365C>T	p.A122V	D	P	D	D	25.2	0.001	0.0015	—	*De novo*
85	1	Chr13(q22..3)	*EDNRB*	c.481A>G	p.K161E	T	P	B	D	23.3	—	—	—	*De novo*
92	1	Chr13(q22..3)	*EDNRB*	c.1018C>G	p.H340D	D	D	D	D	32	—	—	—	*De novo*
111	1	Chr13(q22..3)	*EDNRB*	c.1015C>T	p.L339F	T	D	D	D	23.2	—	—	—	*De novo*

Novel refers to variants that were absent in 200 control subjects; de novo refers to variants that were absent in the parents and 200 control subjects. “NA” means “not applicable.” “—” means none. SIFT D: deleterious (sift ≤ 0.05); T: tolerated (sift > 0.05). Polyphen2_HVAR D: probably damaging (≥0.909); P: possibly damaging (0.447 ≤ pp2_hvar ≤ 0.909); B: benign (pp2_hvar ≤ 0.446). Polyphen2_HDIV_pred D: probably damaging (≥0.957); P: possibly damaging (0.453 ≤ pp2_hdiv ≤ 0.956); B: benign (pp2_hdiv ≤ 0.452). MutationTaster_pred A: disease_causing_automatic; D: disease_causing; N: polymorphism; P: polymorphism_automatic. CADD13_PHRED D: CADD_Phred > 15; InDel is not applicable. “EAS” means East Asian populations.

**Table 4:** Summary of new structure variation (SV) or CNV-detected results of the molecular screening of WS-related genes, including location of mutations and population data.

Subject ID	WS type	Chromosome location	Gene	Mutation	Genomic position		Population frequency		
							1000g_EAS	ExAC_EAS	esp6500si_all
					Start	End			
9	2	Chr3(P13)	*MITF*	c.110_219del110bp	110	219	—	—	—
22	2	Chr3(P13)	*MITF*	Duplication of exons 01 and 02	Promoter2	Exon01	—	—	—
33	2	Chr22(q13.1)	*SOX10*	Large fragment deletions including the whole *SOX10* gene	Promoter2	Exon04	—	—	—

“—” means none.

## Data Availability

The data used to support the findings of this study are available from the corresponding author upon request.

## Supplementary Materials

Supplementary 1 Table S1: clinical signs and symptoms of the 90 WS cases.

Supplementary 2 Table S2: the primer sequences of 6 WS-related genes.

Supplementary 3 Table S3: SNVs detected in the 114 cases.

## References

[1] Dourmishev A. L., Dourmishev L. A., Schwartz R. A., Mph, Janniger C. K. (1999). Waardenburg syndrome. *International Journal of Dermatology*.

[2] Zaman A., Capper R., Baddoo W. (2015). Waardenburg syndrome: more common than you think!. *Clinical Otolaryngology*.

[3] Waardenburg P. (1951). A new syndrome combining developmental anomalies of the eyelids, eyebrows and nose root with pigmentary defects of the iris and head hair and with congenital deafness. *American Journal of Human Genetics*.

[4] Chen H., Jiang L., Xie Z. (2010). Novel mutations of PAX3, MITF, and SOX10 genes in Chinese patients with type I or type II Waardenburg syndrome. *Biochemical and Biophysical Research Communications*.

[5] Wollnik B., Tukel T., Uyguner O. (2003). Homozygous and heterozygous inheritance of PAX3 mutations causes different types of Waardenburg syndrome. *American Journal of Medical Genetics*.

[6] Akutsu Y., Shirai K., Takei A. (2018). A patient with peripheral demyelinating neuropathy, central dysmyelinating leukodystrophy, Waardenburg syndrome, and severe hypoganglionosis associated with a novel SOX10 mutation. *American Journal of Medical Genetics Part A*.

[7] Inoue K., Khajavi M., Ohyama T. (2004). Molecular mechanism for distinct neurological phenotypes conveyed by allelic truncating mutations. *Nature Genetics*.

[8] Falah N., Posey J. E., Thorson W. (2017). 22q11.2q13 duplication including SOX10 causes sex-reversal and peripheral demyelinating neuropathy, central dysmyelinating leukodystrophy, Waardenburg syndrome, and Hirschsprung disease. *American Journal of Medical Genetics Part A*.

[9] Hemmi A., Okamura K., Tazawa R. (2018). Waardenburg syndrome type IIE in a Japanese patient caused by a novel non-frame-shift duplication mutation in the SOX10 gene. *The Journal of Dermatology*.

[10] Wang D., Ren G. F., Zhang H. Z., Yi C. Y., Peng Z. J. (2016). A de novo 2q35-q36.1 deletion incorporating IHH in a Chinese boy (47,XYY) with syndactyly, type III Waardenburg syndrome, and congenital heart disease. *Genetics and Molecular Research*.

[11] Bocángel M. A. P., Melo U. S., Alves L. U. (2018). Waardenburg syndrome: novel mutations in a large Brazilian sample. *European Journal of Medical Genetics*.

[12] Farrer L. A., Arnos K. S., Asher JH Jr (1994). Locus heterogeneity for Waardenburg syndrome is predictive of clinical subtypes. *American Journal of Human Genetics*.

[13] Liu Y., Wang L., Feng Y. (2016). A new genetic diagnostic for enlarged vestibular aqueduct based on next-generation sequencing. *PLoS One*.

[14] Adzhubei I. A., Schmidt S., Peshkin L. (2010). A method and server for predicting damaging missense mutations. *Nature Methods*.

[15] Ng P. C., Henikoff S. (2003). SIFT: predicting amino acid changes that affect protein function. *Nucleic Acids Research*.

[16] Schwarz J. M., Rödelsperger C., Schuelke M., Seelow D. (2010). MutationTaster evaluates disease-causing potential of sequence alterations. *Nature Methods*.

[17] Madden C., Halsted M. J., Hopkin R. J., Choo D. I., Benton C., Greinwald JH Jr (2003). Temporal bone abnormalities associated with hearing loss in Waardenburg syndrome. *Laryngoscope*.

[18] Pingault V., Ente D., Dastot-le Moal F., Goossens M., Marlin S., Bondurand N. (2010). Review and update of mutations causing Waardenburg syndrome. *Human Mutation*.

[19] Liu X. Z., Newton V. E., Read A. P. (1995). Waardenburg syndrome type II: phenotypic findings and diagnostic criteria. *American Journal of Medical Genetics*.

[20] Sánchez-Martín M., Rodríguez-García A., Pérez-Losada J., Sagrera A., Read A. P., Sánchez-García I. (2002). SLUG (SNAI2) deletions in patients with Waardenburg disease. *Human Molecular Genetics*.

[21] Jiang L., Chen H., Jiang W. (2011). Novel mutations in the SOX10 gene in the first two Chinese cases of type IV Waardenburg syndrome. *Biochemical and Biophysical Research Communications*.

[22] Song J., Feng Y., Acke F. R., Coucke P., Vleminckx K., Dhooge I. J. (2015). Hearing loss in Waardenburg syndrome: a systematic review. *Clinical Genetics*.

[23] Zhang H., Chen H., Luo H. (2012). Functional analysis of Waardenburg syndrome-associated PAX3 and SOX10 mutations: report of a dominant-negative SOX10 mutation in Waardenburg syndrome type II. *Human Genetics*.

[24] Morimoto N., Mutai H., Namba K., Kaneko H., Kosaki R., Matsunaga T. (2018). Homozygous EDNRB mutation in a patient with Waardenburg syndrome type 1. *Auris Nasus Larynx*.

[25] Wildhardt G., Zirn B., Graul-Neumann L. M. (2013). Spectrum of novel mutations found in Waardenburg syndrome types 1 and 2: implications for molecular genetic diagnostics. *BMJ Open*.

[26] DeStefano A. L., Cupples L. A., Arnos K. S. (1998). Correlation between Waardenburg syndrome phenotype and genotype in a population of individuals with identified PAX3 mutations. *Human Genetics*.

[27] Chan K. K., Wong C. K. Y., Lui V. C. H., Tam P. K. H., Sham M. H. (2003). Analysis of SOX10 mutations identified in Waardenburg-Hirschsprung patients: differential effects on target gene regulation. *Journal of Cellular Biochemistry*.

[28] Cobaleda C., Pérez-Caro M., Vicente-Dueñas C., Sánchez-García I. (2007). Function of the zinc-finger transcription factor SNAI2 in cancer and development. *Annual Review of Genetics*.

[29] McCallion A. S., Chakravarti A. (2001). EDNRB/EDN3 and Hirschsprung disease type II. *Pigment Cell Research*.

[30] Parichy D. M., Mellgren E. M., Rawls J. F., Lopes S. S., Kelsh R. N., Johnson S. L. (2000). Mutational analysis of endothelin receptor b1 (rose) during neural crest and pigment pattern development in the zebrafish Danio rerio. *Developmental Biology*.

[31] Silan F., Zafer C., Onder I. (2006). Waardenburg syndrome in the Turkish deaf population. *Genetic Counseling*.

[32] Tamayo M. L., Gelvez N., Rodriguez M. (2008). Screening program for Waardenburg syndrome in Colombia: clinical definition and phenotypic variability. *American Journal of Medical Genetics Part A*.

[33] Pardono E., van Bever Y., van den Ende J. (2003). Waardenburg syndrome: clinical differentiation between types I and II. *American Journal of Medical Genetics*.

[34] Sham M. H., Lui V. C., Chen B. L., Fu M., Tam P. K. (2001). Novel mutations of SOX10 suggest a dominant negative role in Waardenburg-Shah syndrome. *Journal of Medical Genetics*.

[35] Bondurand N., Dastot-le Moal F., Stanchina L. (2007). Deletions at the SOX10 gene locus cause Waardenburg syndrome types 2 and 4. *American Journal of Human Genetics*.

[36] Zazo Seco C., Serrão de Castro L., van Nierop J. W. (2015). Allelic mutations of KITLG, encoding KIT ligand, cause asymmetric and unilateral hearing loss and Waardenburg syndrome type 2. *American Journal of Human Genetics*.

