# Supplementary material for: New Genotypes and Phenotypes in Patients with 3 Subtypes of Waardenburg Syndrome Identified by Diagnostic Next-Generation Sequencing
Source: Neural Plast. 2019 Feb 27;2019:7143458. doi: 10.1155/2019/7143458 (PMC6415303; doi:10.1155/2019/7143458)
Supplement: Supplementary 1 — Table S1: clinical signs and symptoms of the 90 WS cases. [file 7143458.f1.pdf]

Table S1. Clinical signs and symptoms of the 90 cases diagnosed with WS.

Subject ID	WS Type		clinical signs and symptoms												
		Heterochromia irides	Hearing loss	Premature white hair	Dystopia canthorum	Freckles	Yellow hair	Amblyopia	Congenital ptosis	Constipation	Congenital megacolon	Pigmentation spots	Narrow palpebral fissures	Hypopigmented skin lesions	Intellectual disability
16	1	-	+	-	+	-	-	-	-	-	-	-	-	-	-
34	1	-	+	-	+	-	-	-	-	-	-	-	-	-	-
35	1	-	+	-	+	-	-	-	-	-	-	-	-	-	-
38	1	+	+	-	+	-	-	-	-	-	-	-	-	-	-
44	1	+	+	-	+	-	-	-	-	-	-	+	-	-	-
45	1	+	+	+	+	-	-	-	-	-	-	-	-	-	-
46	1	-	-	+	+	-	-	-	-	-	-	-	-	-	-
48	1	+	+	-	+	-	-	-	-	-	-	-	-	-	-
53	1	+	+	-	+	-	-	-	-	-	-	-	-	-	-
56	1	+	-	-	+	-	-	-	-	-	-	-	-	-	-
65	1	+	+	-	+	-	-	-	-	-	-	-	-	-	-
66	1	+	+	-	+	-	-	-	-	-	-	-	-	-	-
67	1	+	+	-	+	-	+	-	-	-	-	-	-	-	-
68	1	+	+	-	+	-	+	-	-	-	-	-	-	-	-
69	1	+	-	-	+	-	-	-	-	+	+	-	-	-	-
84	1	+	+	-	+		-	-	-	-	-	-	-	-	-
85	1	+	+	-	+		-	-	-	-	-	-	-	-	-
86	1	+	+	-	+		-	-	-	-	-	-	-	-	-
89	1	+	+	-	+		-	-	-	-	-	-	-	-	-
92	1	+	+	-	-	-	-	-	-	-	-	-	-	-	-
108	1	+	+	-	+	-	-	-	-	-	-	-	-	-	-

109	1	+	+	-	+	-	-	-	-	-	-	-	-	-	-
110	1	-	+	-	+	-	-	-	-	-	-	-	-	-	-
111	1	+	+	-	+	-	-	-	-	-	-	-	-	-	-
112	1	+	+	-	+	-	-	-	-	-	-	-	-	-	-
113	1	+	+	-	+	-	-	-	-	-	-	-	-	-	-
114	1	+	+	-	+	-	-	-	-	-	-	-	-	-	-
1	2	+	+	+	-	+	-	-	-	-	-	-	-	-	-
2	2	+	+	-	-	-	-	-	-	-	-	-	-	-	-
4	2	+	+	-	-	-	-	-	-	-	-	-	-	-	-
5	2	+	+	-	-	-	-	-	-	-	-	-	-	-	-
6	2	+	+	+	-	-	-	-	-	-	-	-	-	-	-
7	2	+	+	-	-	+	-	-	-	-	-	-	-	-	-
8	2	+	+	+	-	+	+	+	-	-	-	-	-	-	-
9	2	+	+	-	-	-	+	-	+	-	-	-	-	-	-
10	2	+	+	-	-	+	-	-	-	-	-	-	-	-	-
11	2	+	+	-	-	-	-	-	-	-	-	-	-	-	-
12	2	+	+	-	-	-	-	-	+	-	-	-	-	-	-
13	2	+	+	-	-	+	-	-	-	-	-	-	-	-	-
14	2	+	+	-	-	-	-	-	-	-	-	-	-	-	-
23	2	+	+	-	-	-	-	-	-	-	-	-	-	-	-
24	2	+	-	-	-	+	-	-	-	-	-	-	-	-	-
28	2	-	+	-	-	+	-	-	-	-	-	+	-	-	-
29	2	-	+	+	-	-	-	-	-	-	-	-	-	-	-
31	2	+	+	-	-	-	-	-	-	-	-	-	-	-	-

33	2	+	+	-	-	-	-	-	-	-	-	-	-	-	-
36	2	+	+	-	-	-	-	-	-	-	-	-	-	-	-
37	2	+	+	-	-	-	-	-	-	-	-	-	+	+	-
40	2	+	+	-	-	-	-	-	-	-	-	-	-	-	-
41	2	+	+	+	-	+	-	-	-	-	-	-	-	-	-
42	2	+	+	+	-	+	-	-	-	-	-	-	-	-	-
43	2	+	+	+	-	+	-	-	-	-	-	-	-	-	-
47	2	+	+	-	-	-	-	-	-	-	-	-	-	-	+
49	2	+	+	-	-	-	-	-	-	-	-	-	-	-	-
51	2	+	+	-	-	-	-	-	-	-	-	-	-	-	-
54	2	+	+	-	-	-	-	-	-	-	-	-	-	-	-
57	2	+	+	-	-	-	-	-	-	-	-	-	-	-	-
58	2	+	+	-	-	-	-	-	-	-	-	-	-	-	-
59	2	+	+	-	-	-	-	-	-	-	-	-	-	-	-
60	2	+	+	-	-	-	-	-	-	-	-	-	-	-	-
61	2	+	+	-	-	-	-	-	-	-	-	-	-	-	-
62	2	-	-	+	-	+	-	-	-	-	-	-	-	-	-
63	2	-	-	+	-	+	-	-	-	-	-	-	-	-	-
64	2	-	+	+	-	+	-	-	-	-	-	-	-	-	-
70	2	+	+	-	-	-	-	-	-	-	-	-	-	+	-
72	2	+	+	-	-	-	-	-	-	-	-	-	-	-	-
76	2	+	+	-	-	-	-	-	-	-	-	-	-	-	-
77	2	+	+	-	-	+	-	-	-	-	-	-	-	-	-
78	2	+	+	-	-	-	-	-	-	-	-	-	-	-	-

79	2	+	+	-	-	-	-	-	-	-	-	-	-	-	-
80	2	+	+	-	-	-	-	-	-	-	-	-	-	+	-
81	2	-	+	+	-	+	-	-	-	-	-	-	-	-	-
82	2	-	+	+	-	+	-	-	-	-	-	-	-	-	-
83	2	+	+	-	-	+	-	-	-	-	-	-	-	-	-
87	2	+	+	-	-	+	-	-	-	-	-	-	-	-	-
88	2	+	+	-	-	-	-	-	-	-	-	-	-	-	-
90	2	+	+	-	-	-	-	-	-	-	-	-	-	-	-
91	2	+	+	-	-	-	-	-	-	-	-	-	-	-	-
94	2	+	+	-	-	-	-	-	-	-	-	-	-	-	-
97	2	+	+	-	-	-	-	-	-	-	-	-	-	-	-
98	2	+	+	-	-	-	-	-	-	-	-	-	-	-	-
100	2	+	+	-	-	-	-	-	-	-	-	-	-	-	-
104	2	+	+	-	-	-	-	-	-	-	-	-	-	-	-
110	2	+	+	-	-	-	-	-	-	-	-	-	-	-	-
17	4	+	+	-	-	-	-	-		+	+	-	-	-	-
18	4	+	+	-	-	-	-	-	-	-	+	-	-	-	-
20	4	+	+	-	-	-	-	-		+	+	-	-	-	-
22	4	+	+	-	-	-	-	+	-	-	+	-	-	-	-
101	4	+	+	-	-	-	-	-	-	-	+	-	-	-	-
102	4	+	+	-	-	-	-	-	-	-	+	-	-	-	-

"+" stands for existence; "-" means no.
