# Supplementary material for: New Genotypes and Phenotypes in Patients with 3 Subtypes of Waardenburg Syndrome Identified by Diagnostic Next-Generation Sequencing
Source: Neural Plast. 2019 Feb 27;2019:7143458. doi: 10.1155/2019/7143458 (PMC6415303; doi:10.1155/2019/7143458)
Supplement: Supplementary 2 — Table S2: the primer sequences of 6 WS-related genes. [file 7143458.f2.pdf]

Table S2. The primer sequences of 6 WS-related genes.

fragment_ name	fragment _size	primer_5'	position	sequence	primer_3'	position	sequence
mSOX10E 04D	275	mSOX10E04 D_5R	chr22:38370038-3 8370059	AGCCACGAGGTAATGTCCAACA	mSOX10E04 D_3F	chr22:38369785-3 8369804	GGCTGGTCGGTGTAGTGTGG
mSOX10E 03A	280	mSOX10E03 A_5R	chr22:38374171-3 8374192	CATTCACGCCAGCCCCTCTGCT	mSOX10E03 A_3F	chr22:38373913-3 8373933	CCTCTCCTGGGTGCCGGTGGT
mSOX10E 03B	200	mSOX10E03 B_5R	chr22:38373977-3 8373996	GTGGGGAGGCCGAGCAAGGT	mSOX10E03 B_3F	chr22:38373797-3 8373822	GTCTCATTGCCATCCAGCCATCTC CT
mSOX10E 02B	274	mSOX10E02 B_5R	chr22:38379777-3 8379796	gcgacatggcggaggAGCAG	mSOX10E02 B_3F	chr22:38379523-3 8379541	ATGGGCACCAGCGTCCAGT
mSOX10E 01A	261	mSOX10E01 A_5R	chr22:38380552-3 8380574	CGGCTCAGGCTCAGTCGCTCAGT	mSOX10E01 A_3F	chr22:38380314-3 8380332	GCGGGCCAGGGAGGAGCAG
mSOX10E 02A	209	mSOX10E02 A_5R	chr22:38379933-3 8379954	GGGCTGTGCCCACGTCCTGTCT	mSOX10E02 A_3F	chr22:38379746-3 8379766	CCACGGGGCTCAGCTCCACCT
mSOX10E 04C	243	mSOX10E04 C_5R	chr22:38369849-3 8369870	GTCTCACCACCTGGTGTGGATG	mSOX10E04 C_3F	chr22:38369628-3 8369649	GAAGGCCGAGTAGAGGCCAGAG
mSOX10E 02C	277	mSOX10E02 C_5R	chr22:38379550-3 8379571	AGGCCGTCAGCCAGGTGCTCAG	mSOX10E02 C_3F	chr22:38379295-3 8379316	GCCGCCTCGGCTACCCTGAATC
mSOX10E 04B	275	mSOX10E04 B_5R	chr22:38369694-3 8369715	CCGCCCCCAGTTTGACTACTCT	mSOX10E04 B_3F	chr22:38369441-3 8369460	GAACAGGGCACACAGGCTGG
mSNAI2E 01A	246	mSNAI2E01 A_5R	chr8:49834019-49 834050	CTGTGATTGGATCTTTCTTGCAAAA GAGAGGA	mSNAI2E01 A_3F	chr8:49833805-49 833826	ACCAGGAAGGAGCGCGGCATCT
mSNAI2E 01B	269	mSNAI2E01 B_5R	chr8:49833857-49 833878	GTCCTCCCGCCGGACCGTTATC	mSNAI2E01 B_3F	chr8:49833610-49 833641	TCCCTTTCAGGACACTGTTAAAAA GGAAAACA

mSNAI2E03A	235	mSNAI2E03A_5R	chr8:49831528-49831549	AGGGGAGAAGCCTTTTTCTTGC	mSNAI2E03A_3F	chr8:49831315-49831338	GCTTCGGAGTGAAGAAATGCATTC
mSNAI2E02C	277	mSNAI2E02C_5R	chr8:49833013-49833036	TGCCTTAATCTTCTCTCCCTCCCT	mSNAI2E02C_3F	chr8:49832760-49832781	CTAATGGGGCTTTCTGAGCCAC
mSNAI2E03B	251	mSNAI2E03B_5R	chr8:49831705-49831740	TAAATGCAATATTCAAGTTTATCTTAAAAATTCAGG	mSNAI2E03B_3F	chr8:49831490-49831515	AGATTTGACCTGTCTGCAAATGCTCT
mSNAI2E02B	257	mSNAI2E02B_5R	chr8:49832800-49832819	TCCCCCTCCTCCATCTGACA	mSNAI2E02B_3F	chr8:49832563-49832582	CGCCCAGGCTCACATATTCC
mSNAI2E02A	247	mSNAI2E02A_5R	chr8:49832621-49832638	CAGCTGCACTGCGATGCC	mSNAI2E02A_3F	chr8:49832392-49832409	ACAGCCAGCCCAGGGCTT
mMITFE07A	285	mMITFE07A_5F	chr3:70005561-70005588	CATGGCACTGTTACTAATAGCCTTTCCT	mMITFE07A_3R	chr3:70005814-70005845	TGAGACTAACCAAAAGAAAGGTGTAAGAAGAA
mMITFE01A	255	mMITFE01A_5F	chr3:69985655-69985680	AAAAAGGCCCTTATGTGAACGTTTTT	mMITFE01A_3R	chr3:69985878-69985909	CACCTGATAGTGATTATATTCTAGCATTTCCA
mMITFE05A	208	mMITFE05A_5F	chr3:69998153-69998184	ATGGGAATTGTTCAACAGTTAATTTCTGTTAC	mMITFE05A_3R	chr3:69998333-69998360	ACAGAAATCTGCAGTAAGAGGCACATTT
mMITFE04A	265	mMITFE04A_5F	chr3:69990342-69990377	AGTTTATTTATTTTTGTCTCTCTTCTCTTACCCTTT	mMITFE04A_3R	chr3:69990571-69990606	CCATTTTACTATTAATACATTTAATCAGTGAACCAG
mMITFE01B	279	mMITFE01B_5F	chr3:69985798-69985829	AAATTGGAATTATAGAAAGTAGAGGGAGGGAT	mMITFE01B_3R	chr3:69986043-69986076	GTTCAAAACTACATTGAATTACTTCTTATGGCAT
mMITFE08A	230	mMITFE08A_5F	chr3:70008379-70008408	GCCTCAAATCCTAAAAATATCTGTTTTCCT	mMITFE08A_3R	chr3:70008579-70008608	AAAAAGCAGTAAACTTTACACAACTCAGGC
mMITFE09B	285	mMITFE09B_5F	chr3:70014155-70014176	CAACTCTCGATCTCACGGATGG	mMITFE09B_3R	chr3:70014418-70014439	AAGGAAGCAGTTTGTGCGAATG
mMITFE0	287	mMITFE06A	chr3:70000914-70	TGCAACTTCAAACAGTTCCAACTT	mMITFE06A	chr3:70001167-70	GGAAAATATATACCTTGGTTTCTG

6A		_5F	000941	CTAA	_3R	001200	TCAAATACAA
mMITFE09A	275	mMITFE09A_5F	chr3:70013949-70013972	tcctctgtgctctgcctatttcag	mMITFE09A_3R	chr3:70014203-70014223	GGTTGGCCTCAGTCCCAGTTC
mMITFE02B	273	mMITFE02B_5F	chr3:69987093-69987112	CCATGTCCAAACCAGCCTGG	mMITFE02B_3R	chr3:69987344-69987365	AGACAACAGGCTGGAATGCCAC
mMITFE02A	277	mMITFE02A_5F	chr3:69986906-69986929	AAGGAAGAGCCGTCTGAAACTCAC	mMITFE02A_3R	chr3:69987159-69987182	GTTAAGCGTAAGCATAGCCATGGG
mMITFE03A	251	mMITFE03A_5F	chr3:69988171-69988194	TCAGCTTTGTGTGAACAGGTCATT	mMITFE03A_3R	chr3:69988397-69988421	TGCTTAAGTTTTCAGGAAGGTGTGA
mEDNRBE03A	245	mEDNRBE03A_5R	chr13:78477770-78477791	GGGCATCTTATGAAACCCCCTA	mEDNRBE03A_3F	chr13:78477547-78477578	GAATTGCACAGTTTATTTTCTAAGTAACATGG
mEDNRBE08E	284	mEDNRBE08E_5R	chr13:78471784-78471805	TGCATTTTTCGGACACTGGAAA	mEDNRBE08E_3F	chr13:78471522-78471545	TCTTTTGGGATAGCCTTTCAAACA
mEDNRBE02A	211	mEDNRBE02A_5R	chr13:78492350-78492369	CTGGGGATCATCGGGAACTC	mEDNRBE02A_3F	chr13:78492159-78492178	TCATCTCCCCGTCTCCAACC
mEDNRBE08J	287	mEDNRBE08J_5R	chr13:78470781-78470805	AGCAAAACACAGTGCAATGTTCTCA	mEDNRBE08J_3F	chr13:78470519-78470546	TGAAATTAAAGAACAAGTTTGTGGGTGA
mEDNRBE08A	260	mEDNRBE08A_5R	chr13:78472510-78472531	GGAGGAAGAGTTGGGAAAGGTG	mEDNRBE08A_3F	chr13:78472272-78472297	CATTTTGTTTTAATGACTTCGGTCCA
mEDNRBE08F	270	mEDNRBE08F_5R	chr13:78471558-78471587	TTTACATTCAGTTAAGATCAAACCTCACAA	mEDNRBE08F_3F	chr13:78471318-78471343	GGCATCTCTCCATCGTAAAGCTATGA
mEDNRBE08B	234	mEDNRBE08B_5R	chr13:78472337-78472366	TCCGTTCCAGTAATAAATACAGCTCATCTT	mEDNRBE08B_3F	chr13:78472133-78472155	TCCCACTGATTTTCTTTCCATGC
mEDNRBE08G	288	mEDNRBE08G_5R	chr13:78471345-78471362	ATGAGGGCAGGCCCTGTG	mEDNRBE08G_3F	chr13:78471075-78471100	CCATGTCAAGCAAACTTTTCTATTGG

mEDNRBE07A	251	mEDNRBE07A_5R	chr13:78474154-78474185	CCTTACAAAAGCACAGAAGCTACAATGACTAC	mEDNRBE07A_3F	chr13:78473935-78473966	TGAAAGCTTATATTTGAGCCATATTACAAAGA
mEDNRBE04B	237	mEDNRBE04B_5R	chr13:78477622-78477649	TGTGCTCTGAGTATTGACAGGTAGAAGC	mEDNRBE04B_3F	chr13:78477413-78477440	CCCAAATCAAAACAATTTCTACTGCTGT
mEDNRBE08I	290	mEDNRBE08I_5R	chr13:78470989-78471015	TGTGCGTTCCTGCCTAATTTTTATATC	mEDNRBE08I_3F	chr13:78470726-78470755	CATATTTTAAGACCGAGTTAAAGCTCTTGG
mEDNRBE01A	252	mEDNRBE01A_5R	chr13:78549679-78549702	gaggcagctccttaggtgacttaa	mEDNRBE01A_3F	chr13:78549451-78549471	GGAGTGGGCAAGTTCCAAAGA
mEDNRBE02C	267	mEDNRBE02C_5R	chr13:78492742-78492763	CTAGGCTCTGAAACTGCGGAGC	mEDNRBE02C_3F	chr13:78492497-78492514	ACCTCCGCAGGTGCCAAC
mEDNRBE04A	259	mEDNRBE04A_5R	chr13:78477449-78477476	TGGAGTAGAATTAAAGGAATTGGGGTTC	mEDNRBE04A_3F	chr13:78477218-78477241	GGGGAACAGGGGAAAAATAGCTAA
mEDNRBE05A	245	mEDNRBE05A_5R	chr13:78475353-78475387	TCTTACAGAGGAGTATTAATCGTAAAAATTCTCTC	mEDNRBE05A_3F	chr13:78475143-78475175	TCATAATTATAAATTCAACCACGAGTTATCAAA
mEDNRBE06A	220	mEDNRBE06A_5R	chr13:78474799-78474826	GCCATCTTTTAAGGGTCAAACTATGGAT	mEDNRBE06A_3F	chr13:78474607-78474634	TTCTGAGTGGCATTTATTTACAAAACCA
mEDNRBE08D	283	mEDNRBE08D_5R	chr13:78472000-78472030	AATGAGCTCACTCAGAATTTCTATTCTTTCT	mEDNRBE08D_3F	chr13:78471748-78471772	TGCTCTTTCTGTTACTCCCTCCTGA
mEDNRBE02B	279	mEDNRBE02B_5R	chr13:78492555-78492580	AGAGATAATGACGCCACCCACTAAGA	mEDNRBE02B_3F	chr13:78492302-78492321	GGACCGTTTCGCATGCACTT
mEDNRBE08H	290	mEDNRBE08H_5R	chr13:78471229-78471249	CCTCTCTCACCCATGCTGTGG	mEDNRBE08H_3F	chr13:78470960-78470984	CAAGCTATCCTAAGGCACTTTGCTT
mEDNRBE02D	267	mEDNRBE02D_5R	chr13:78492953-78492972	CGGGGGAAGAAAAACAGCTG	mEDNRBE02D_3F	chr13:78492706-78492727	CATGCTGCTACCTGCTCCAGAA
mEDNRB	237	mEDNRBE0	chr13:78472156-7	AGCTACATATGACATTTTATGAGC	mEDNRBE0	chr13:78471951-7	TTCTTCCTTTATATCAGAGTCCCAT

E08C		8C_5R	8472187	TGTTTACG	8C_3F	8471979	TGAT
mPAX3E08A	257	mPAX3E08A_5R	chr2:223066809-223066830	GTCTGGAACCTACCACCACGGT	mPAX3E08A_3F	chr2:223066574-223066603	AATCTTGCCTCTAATTCACCTTTCTTAAGG
mPAX3E01A	259	mPAX3E01A_5R	chr2:223163370-223163391	TCGCCTTTCCGTTTCGCCTTCA	mPAX3E01A_3F	chr2:223163133-223163154	GTGCCCAGGGAATGGGCTTCCT
mPAX3E02A	193	mPAX3E02A_5R	chr2:223161825-223161842	TCGTGGAGATGGCCCACC	mPAX3E02A_3F	chr2:223161650-223161669	AGGGGACCACAGTCTGGGAG
mPAX3E08B	277	mPAX3E08B_5R	chr2:223067026-223067049	GGGTTCATAGTCTCCTGGACAGCT	mPAX3E08B_3F	chr2:223066773-223066800	TTCATATGGTCTAGTCTCTGACTGCAGC
mPAX3E02B	227	mPAX3E02B_5R	chr2:223161968-223161995	TTCTTTGCTTTTTATTTTCCTCCAGTGA	mPAX3E02B_3F	chr2:223161769-223161786	GACGCAGCCGTGGGACAC
mPAX3E07A	243	mPAX3E07A_5R	chr2:223085005-223085028	CTCCAAGCACTGTACACCAAAGCA	mPAX3E07A_3F	chr2:223084786-223084813	ACTACTGCCTCAGGGAATTGAATATTTG
mPAX3E06B	203	mPAX3E06B_5R	chr2:223086066-223086087	GTGCAAGATGGAGGAAGCAAGC	mPAX3E06B_3F	chr2:223085885-223085906	CACCAGAGAAATCGCCTGGAAG
mPAX3E03A	238	mPAX3E03A_5R	chr2:223160410-223160427	ACCTGGCCCAGGGTACCG	mPAX3E03A_3F	chr2:223160190-223160211	GCCGGGGTAATAGCGACTGACT
mPAX3E07B	277	mPAX3E07B_5R	chr2:223085186-223085213	TTAAGAAACGCAGTTTGAAGTGAACATT	mPAX3E07B_3F	chr2:223084937-223084956	GCTGGAAAATCCATGCCTGG
mPAX3E06A	231	mPAX3E06A_5R	chr2:223086203-223086225	TCTTGCATCTATTTGCCCTTTCA	mPAX3E06A_3F	chr2:223085995-223086012	AGGTCGGCATGGCAGTGG
mPAX3E05B	269	mPAX3E05B_5R	chr2:223097176-223097199	ACATTGGGAATGCACTGTAGACCA	mPAX3E05B_3F	chr2:223096931-223096958	GCTGTTTCCTCTTTAGTGGTAAATCTGG
mPAX3E04A	223	mPAX3E04A_5R	chr2:223159040-223159057	GTGCGCTCCCTTGCTTCC	mPAX3E04A_3F	chr2:223158835-223158854	ATGCCCTCCAAGTCACCCAG

mPAX3E05A	279	mPAX3E05A_5R	chr2:223097001-223097028	GATGTTTTGTTCTTCTGTGCTTTTAGCC	mPAX3E05A_3F	chr2:223096750-223096775	TTTCCTGTCTGGACTGAAGTAGGACA
mEDN3E04A	271	mEDN3E04A_5F	chr20:57897368-57897389	GAAAAACCAGCCACAGGGAAAG	mEDN3E04A_3R	chr20:57897619-57897638	CCACCAAAGGCACCATTGCT
mEDN3E02A	242	mEDN3E02A_5F	chr20:57876409-57876428	GGGTGTTGAGGGGTCTGCAC	mEDN3E02A_3R	chr20:57876633-57876650	CAGGGCTTCCAGGGCTTG
mEDN3E03A	260	mEDN3E03A_5F	chr20:57896022-57896039	GAGCAGGGAGGCCTGGGT	mEDN3E03A_3R	chr20:57896258-57896281	TGCAAATGAGTGAAACTGGAGGAG
mEDN3E01A	185	mEDN3E01A_5F	chr20:57875829-57875847	GTCCTCCTGGTCCGGTGCT	mEDN3E01A_3R	chr20:57875995-57876013	CGCGACGCACATCTTCTCC
mEDN3E02B	250	mEDN3E02B_5F	chr20:57876607-57876624	TGTGGCCCCGACAGCACT	mEDN3E02B_3R	chr20:57876835-57876856	GGAGTGAGGTCCTCCTGGGAAT
mEDN3E05A	272	mEDN3E05A_5F	chr20:57899287-57899308	TGGGAAGACGTTCCCTTTTTCA	mEDN3E05A_3R	chr20:57899537-57899558	ATGACAGAAGCCTCCCGAGACC
SOX10_E04E	287	SOX10_E04E_5F2	chr22:38370017-38370037	CAGCCACATCAAAGGTCTCCA	SOX10_E04E_3R2	chr22:38370285-38370303	CCGCTGCCTGAAGGTGAAC
