# Supplementary material for: New Genotypes and Phenotypes in Patients with 3 Subtypes of Waardenburg Syndrome Identified by Diagnostic Next-Generation Sequencing
Source: Neural Plast. 2019 Feb 27;2019:7143458. doi: 10.1155/2019/7143458 (PMC6415303; doi:10.1155/2019/7143458)
Supplement: Supplementary 3 — Table S3: SNVs detected in the 114 cases. [file 7143458.f3.pdf]

Table S3. SNVs detected in the 114 cases.

Subject ID	WS Type	Gene	Mutation	AA Change	Records in HGMD	Report
16	1	*PAX3*	c.586G>A	p.A196T	Waardenburg syndrome	Y
34	1	*PAX3*	c.784C>T	p.R262*	Waardenburg syndrome	Y
35	1	*PAX3*	c.784C>T	p.R262*	Waardenburg syndrome	Y
38	1	*PAX3*	c.232G>A	p.V78M	Waardenburg syndrome	Y
44	1	*PAX3*	c.793+1G>A	-	Waardenburg syndrome 1	Y
45	1	-	-	-	-	-
46	1	-	-	-	-	-
48	1	*EDNRB*	c.245G>A	p.R82Q;-	Waardenburg syndrome	Y
53	1	*PAX3*	c.452-2A>G	-	Waardenburg syndrome	Y
56	1	-	-	-	-	-
65	1	*PAX3*	c.793-3T>G	NA	-	N
		EDNRB	c.469A>G	p.I157V	Waardenburg syndrome 4	Y
66	1	*PAX3*	c.793-3T>G	NA	-	N
		EDNRB	c.469A>G	p.I157V	Waardenburg syndrome 4	Y
67	1	*MITF*	c.1022_1023delTC	p.L341Rfs*18	Waardenburg syndrome 1	Y
68	1	*MITF*	c.1022_1023delTC	p.L341Rfs*18	Waardenburg syndrome 1	Y
69	1	-	-	-	-	-
84	1	*PAX3*	c.72delG	p.G24fs	Waardenburg syndrome	Y
85	1	*EDNRB*	c.553G>A	p.V185M	Hirschsprung disease	Y

		EDNRB	c.481A>G	p.K161E	-	N
86	1	*PAX3*	c.185T>C	p.M62T	Waardenburg syndrome	Y
89	1	*PAX3*	c.128G>T	p.G43V	Waardenburg syndrome	Y
92	1	*EDNRB*	c.1018C>G	p.H340D	-	N
108	1	*EDNRB*	c.245G>A	p.R82Q;-	Waardenburg syndrome	Y
109	1	*PAX3*	c.808C>G	p.R270G	-	N
110	1	*MITF*	c.1260G>C	p.*420Y	Waardenburg syndrome 2	Y
111	1	*EDNRB*	c.1015C>T	p.L339F	-	N
112	1	*PAX3*	c.152T>G	p.L51R		N
113	1	*PAX3*	c.803G>T	p.S268I	-	N
			c.801delT	p.F267LfsX17	-	N
114	1	*PAX3*	c.803G>T	p.S268I	-	N
			c.801delT	p.F267LfsX17	-	N
1	2	*MITF*	c.650 G>T	p.R217I	Waardenburg syndrome 2	Y
2	2	*PAX3*	c.238C>G	p.H80D	Waardenburg syndrome 1	Y
4	2	*PAX3*	c.556delC	p. H186Tfs*7	Waardenburg syndrome 1	Y
5	2	-	-	-	-	-
6	2	*PAX3*	c.808 C>T	p.R270C	Waardenburg syndrome	Y
7	2	*MITF*	c.642_650delAAG	NA	-	N
8	2	*MITF*	c.642_650delAAG	NA	-	N
9	2	-	-	-	-	-
10	2	*MITF*	c.575delC	p.T192Kfs*20	Waardenburg syndrome 2	Y
11	2	*SOX10*	c.126_127 GC>TT	p.R43*	Waardenburg syndrome 2	Y

12	2	*SOX10*	c.113delG	p.G38Afs*71	Waardenburg syndrome 2	Y
13	2	*MITF*	c..642_650 delAAG	NA	-	N
14	2	*SOX10*	c.743_744delAG	p. E248Afs*32	Waardenburg syndrome 2	Y
23	2	*MITF*	c.763 C>T	p. R255*	Waardenburg syndrome	Y
24	2	*MITF*	c.763 C>T	p. R255*	Waardenburg syndrome	Y
28	2	*MITF*	c.328 C>T	p.R110*	Waardenburg syndrome	Y
29	2	*MITF*	c.328 C>T	p.R110*	Waardenburg syndrome	Y
31	2	-	-	-	-	-
33	2	-	-	-	-	-
36	2	-	-	-	-	-
37	2	-	-	-	-	-
40	2	*MITF*	c.944_946del	NA	Waardenburg syndrome	Y
41	2	*MITF*	c.944_946del	NA	Waardenburg syndrome	Y
42	2	*MITF*	c.944_946del	NA	Waardenburg syndrome	Y
43	2	*MITF*	c.944_946del	NA	Waardenburg syndrome	Y
47	2	-	-	-	-	-
49	2	*PAX3*	c.808C>G	,p.R270G	-	N
51	2	*PAX3*	c.117C>A	p.N39K	-	N
54	2	*PAX3*	c.452-2A>G	-	Waardenburg syndrome	Y
57	2	-	-	-	-	-
58	2	-	-	-	-	-
59	2	-	-	-	-	-
60	2	-	-	-	-	-
61	2	*MITF*	c.1013+1G>A	NA	Waardenburg syndrome 2	Y

62	2	*MITF*	c.1013+1G>A	NA	Waardenburg syndrome 2	Y
63	2	*MITF*	c.1013+1G>A	NA	Waardenburg syndrome 2	Y
64	2	*MITF*	c.1013+1G>A	NA	Waardenburg syndrome 2	Y
70	2	*SOX10*	c.698-52_698-42delCTCTAACCTGC	-	Hirschsprung disease	Y
72	2	*EDN3*	c.49G>A	p.A17T	Hirschsprung disease	Y
		MITF	c.1066C>T	p.R356X	Waardenburg syndrome	Y
76	2	*EDN3*	c.49G>A	p.A17T	Hirschsprung disease	Y
		MITF	c.944_946del	NA	Waardenburg syndrome	Y
		SNAI2	c.365C>T	p.A122V	-	N
		SOX10	c.122G>T	p.G41V	-	N
77	2	*MITF*	c.944_946del	NA	Waardenburg syndrome	Y
78	2	-	-	-	-	-
79	2	-	-	-	-	-
80	2A	*MITF*	c.943C>T	p.R214*	Waardenburg syndrome 2A	Y
81	2	*MITF*	c.649C>T	p. R217*	Waardenburg syndrome	Y
82	2	*MITF*	c.649C>T	p. R217*	Waardenburg syndrome	Y
83	2	*MITF*	c.575delC	p.T192fs	Waardenburg syndrome 2	Y
87	2	-	-	-	-	-
88	2	*SOX10*	c.422T>C	p.L141P	Waardenburg syndrome	Y
90	2	*PAX3*	c.238C>G	p.H80D	Waardenburg syndrome 1	Y
91	2	*PAX3*	c.556delC	p.H186fs	Waardenburg syndrome 1	Y
94	2	*SOX10*	c.1063C>T	p.Q355*	Waardenburg syndrome 4	Y
97	2	*SOX10*	c.743_744del	p.248_248del;	Waardenburg syndrome 2	Y

98	2	*PAX3*	c.586G>A	p.A196T	Waardenburg syndrome	Y
100	2	*SOX10*	c.254G>A	p.W85*	Waardenburg syndrome 4	Y
104	2	*EDN3*	c.49G>A	p.A17T	Hirschsprung disease	Y
		PAX3	c.784C>T	p.R262*	，Waardenburg syndrome	Y
110	2	-	-	-	-	-
17	4	*SOX10*	c.254G>A	p.W85*	Waardenburg syndrome 4	Y
		SNAI2	c.230C>G	p.S77C	-	N
18	4	*SOX10*	c.254G>A	p.W85*	Waardenburg syndrome 4	Y
20	4	*SOX10*	c.122G>T	p.G41V	-	N
			c.698-2A>T	-	Waardenburg syndrome 4	Y
22	4	*SOX10*	c.1063 C>T	p.Q355X	Waardenburg syndrome 4	Y
101	4	*EDNRB*	c.245G>A	p.R82Q;	Waardenburg syndrome 4	Y
		SOX10	c.230C>G	p.S77C;	-	N
102	4	*SOX10*	c.127C>T	p.R43X	-	N
3	NA	*PAX3*	c.238C>G	p.H80D	Waardenburg syndrome 1	Y
15	NA	*SOX10*	c.743_744delAG	p. E248Afs*32	Waardenburg syndrome 2	Y
21	NA	*SOX10*	c.122G>T	p.G41V	-	N
25	NA	*MITF*	c.763 C>T	p. R255*	Waardenburg syndrome	Y
26	NA	*MITF*	c.763 C>T	p. R255*	Waardenburg syndrome	Y
27	NA	*MITF*	c.763 C>T	p. R255*	Waardenburg syndrome	Y
30	NA	-	-	-	-	-
32	NA	-	-	-	-	-
55	NA	*PAX3*	c.452-2A>G	-	Waardenburg syndrome	Y
71	NA	*EDN3*	c.49G>A	p.A17T	Hirschsprung disease	Y

		MITF	c.1066C>T	p.R356X	Waardenburg syndrome	Y
73	NA	*MITF*	c.1066C>T	p.R255*	Waardenburg syndrome	Y
74	NA	*MITF*	c.1066C>T	p.R255*	Waardenburg syndrome	Y
75	NA	*MITF*	c.1066C>T	p.R255*	Waardenburg syndrome	Y
78	NA	*MITF*	c.944_946del	NA	Waardenburg syndrome	Y
79	NA	*MITF*	c.944_946del	NA	Waardenburg syndrome	Y
95	NA	*SOX10*	c.113delG	p.G38fs	Waardenburg syndrome 2	Y
96	NA	*SOX10*	c.113delG	p.G38fs	Waardenburg syndrome 2	Y
103	NA	*SNAI2*	c.365C>T	p.A122V;	-	N
105	NA	*EDN3*	c.49G>A	p.A17T	Waardenburg syndrome	Y
		PAX3	c.784C>T	p.R262*	Waardenburg syndrome	Y

"-" in gene and mutation means negative result; "-" in AA Change means not applicable; "-" in Records in HGMD means no. "NA" represents " not applicable ". "Y" stands for "Yes"; "N" for "No".
